# Supplementary material for: Identification of CDPK Gene Family in Solanum habrochaites and Its Function Analysis under Stress
Source: Int J Mol Sci. 2022 Apr 11;23(8):4227. doi: 10.3390/ijms23084227 (PMC9031491; doi:10.3390/ijms23084227)
Supplement: Supplementary file 1 [file ijms-23-04227-s001.zip › ijms-1662611-supplementary.pdf]

Primer name	Forward primer（5′-3′）(Tm)	Reverse primer（5′-3′）(Tm)
CDPK1	CAACCCCTCGTCACCATGAA(57.45)	GGCGTAGTCGATACCGGAAG(59.50)
CDPK2	CGGTGCAAAGCTACCACTTG(57.45)	TCAAAGTTTTCGACAACGCCC(55.61)
CDPK3	CAGATGGAGCAAAAGACTTGGT(55.81)	GGTGTGTCTGGTGCTTCTCC(59.50)
CDPK4	AGAGGCCACTATACGGAAAGG(57.57)	GCCTGTGACACATCTGAACG(57.45)
CDPK5	GGAAGAGGAAATCGCTGGCT(57.45)	CCGCAGCCTTCATCAAATCG(57.45)
CDPK6	TGATGGAGCTTTGTGTGGGT(55.40)	CTAGCGAGTTCAGCTGCCTT(57.45)
CDPK7	ATGGCAACAGAGCCACACTT(55.40)	ATAATGCGCCGGACTAGGAC(57.45)
*CDPK*8	TGGAAATCGTGTGGCTGTCA(55.40)	GGTCTAACAGTTCTCCGCCC(59.50)
CDPK9	CAAGGAGGGAGCTGAAGTGG(59.50)	GGAGACGGTGGAGGAAATGG(59.50)
*CDPK*10	GGATGGGCAAATCAGTTACGAA(55.81)	AAGCTGGAGAGACCCATCCT(57.45)
*CDPK*11	GGGAACCATGGCCTCAAGTC(59.50)	TCCAGGGGTGATTTACCAAGG(57.57)
*CDPK*12	AGGCCCTTGCAAAGGATCTC(57.45)	GCCCATCCGTGTTACTGTCA(57.45)
*CDPK*13	ACTGTTACTGTGCAGGCTGG(57.45)	TCCAAACTGACCCCTACCCA(57.45)
*CDPK*14	TGAAGTCTTGTGCCATCGCT(55.40)	AGCAATGACCCTCAAAGCCA(55.40)
*CDPK*15	TGAAGCCGACATGGGTTCTC(55.45)	ACCAAATTGGCCTTGCCCTA(55.40)
*CDPK*16	ATGAAGCCCTCCAACATCCG(57.45)	CGCGATAACCTTAAGCGCGA(57.45)
*CDPK*17	CGCCTCAAGCAATTCTCAGC(57.45)	CCTACTCGCCTCAAACCCTC(59.50)
*CDPK*18	CCAAGAGCGCAAGTAGGACA(57.45)	TCGCCAGATGGATCGTTCAC(57.45)
CDPK19	TTTTGGGCTGAGACGGACAA(55.40)	CGCGCTTTAGGATCCCTATCA(57.57)
CDPK20	GATGGAGTTGTGTGGAGGGG(59.50)	GAGATCACGATGCAACACGC(57.45)
CDPK21	AGCTTATCTTCCGACCAAGCC(57.57)	ACGGCGTTGAAGTAGTAGCG(57.45)
CDPK22	TTACATCGAGCCGGATGAGC(57.45)	GCCTTTCTCCAGTCTGTCCC(59.50)
CDPK23	AGGAGCATTATGATTTGGGAGAAA(54.44)	TTCCCCAGTTGCTTTTTCAACA(53.95)
CDPK24	AAGGAAGCAAAAGATCAACAATCA(52.73)	AGCGTAGATGGCAAGCAGTT(57.45)

CDPK25	GAGGTGAATTGGGTGGAGATG(57.57)	AGGATGCTTAGGCAAATGCT(53.35)
CDPK26	TCCTGCGAAAGCGTTATGGT(55.40)	GAGGCACACCGCTAAGAAGT(57.45)
CDPK27	ACTCAGATCCTTGGCCGTTG(57.45)	AGCGACTCCATTTTCGCAGA(55.40)
CDPK28	CCCGGGGAACGGTTTAATGA(57.45)	TCTGCCCAGAAAGGTGGAAC(57.45)
CDPK29	AACAATAGCAAAAAGGAAGCTGG(54.20)	AAAAGTTCACCACCAGCACAT(53.66)
CDPK30	AATCAGAACCTTGGCCTCAGA(55.61)	AGGATCCCTGGTGAGCATTTTCT(57.77)
CDPK31	AGCTGACCAGGACAATGACG(57.45)	TCGTTCTCGTGAGTTTCTGC(55.40)
CDPK32	GGGTTGTTGCTCAAAGGCAG(57.45)	TTGGTGATGCATGTGGTGGA(55.40)
CDPK33	CCTTGGCTCAAGGTAGGTGG(59.50)	ACATTGATTTGAGCCCCCGA(55.40)
**ACTIN**	TGTCCTATTTACGAGGGTTATGC(53.35)	CAGTTAAATCACGACCAGCAAGAT(55.49)

Table S1. qRT-PCR primer sequence.

Table S2. Gene replication in *CDPK* gene family of *Solanum habrochaites.*

Duplication gene pairs	Ka	Ks	Ka/Ks	Duplication character	Divergence time（Mya）
ShCDPK1:*ShCDPK27*	0.050826	0.877969	0.0578904	Tandem repeat	77.6
ShCDDPK4:*ShCDPK28*	0.0565091	0.862348	0.0655294	Tandem repeat	76.2
ShCDPK5:*ShCDPK23*	0.142765	1.30348	0.109526	Tandem repeat	115.2
ShCDPK6:*ShCDPK24*	0.118572	0.868479	0.136529	Tandem repeat	76.8
ShCDPK6:*ShCDPK26*	0.978784	1.07424	0.91114	Tandem repeat	95.0
ShCDPK24:*ShCDPK26*	0.964807	1.1343	0.850573	Tandem repeat	100.3
ShCDPK30:*ShCDPK19*	0.0609658	0.84017	0.0725637	Tandem repeat	74.3
ShCDPK20:*ShCDPK33*	0.0784101	0.74923	0.104654	Tandem repeat	66.2
ShCDPK7:*ShCDPK9*	0.215039	0.0932819	2.30526	Tandem repeat	8.2
ShCDPK8:*ShCDPK12*	0.073883	0.725424	0.101848	Tandem repeat	64.1
ShCDPK15:*ShCDPK17*	0.0579405	0.582849	0.0994092	Tandem repeat	51.5
				Tandem repeat	

Table S3. Analysis of cis acting elements of *CDPK* gene family in *Solanum habrochaites.*

Gene name	Light	ABRE	TCA	ARE	CGTCA-motif	TC-richrepeats	LTR	MBS	TGA-element	P-BOX
ShCDPK1	√	√	√	-	√	√	-	-	-	-
ShCDPK2	√	√	√	-	√	√	-	-	-	-
ShCDPK3	√	-	-	√	√	√	-	-	√	-
ShCDPK4	√	-	-	√	-	-	-	-	√	-
ShCDPK5	√	√	√	√	-	√	-	-	-	√

ShCDPK6	√	√	-	√	√	√	√	√	-	√
ShCDPK7	√	√	-	√	√	-	√	-	-	-
ShCDPK8	√	√	-	√	√	-	√	-	√	√
ShCDPK9	√	-	-	√	√	-	-	-	√	-
ShCDPK10	√	√	√	-	-	√	-	√	√	-
ShCDPK11	√	√	√	-	√	√	-	√	√	-
ShCDPK12	√	√	-	-	√	-	-	-	-	-
ShCDPK13	√	√	-	-	√	√	-	-	√	-
ShCDPK14	√	√	√	√	-	√	√	-	-	√
ShCDPK15	√	√	-	√	√	√	-	√	-	-
ShCDPK16	√	√	-	√	-	-	-	-	√	-
ShCDPK17	√	-	-	√	-	√	-	√	-	-
ShCDPK18	√	-	-	-	√	-	√	-	-	-
ShCDPK19	√	√	-	-	-	√	-	√	-	-
ShCDPK20	√	-	-	√	√	√	-	√	-	-
ShCDPK21	√	√	√	√	-	√	√	-	-	-
ShCDPK22	√	-	√	-	√	√	-	-	√	-
ShCDPK23	√	√	-	√	√	-	√	-	-	-
ShCDPK24	√	√	√	-	-	-	√	-	-	-
ShCDPK25	√	√	√	-	-	-	-	-	-	√
ShCDPK26	√	-	-	√	-	-	-	√	-	-
ShCDPK27	√	√	-	√	√	√	√	-	-	-
ShCDPK28	√	-	-	√	-	-	-	-	-	√
ShCDPK29	√	-	√	-	√	-	-	-	-	-
ShCDPK30	√	√	-	-	√	√	√	-	-	-
ShCDPK31	√	√	-	-	√	√	-	√	-	-
ShCDPK32	√	√	√	-	-	√	-	-	-	-
ShCDPK33	√	√:	√	√	-	-	√	-	√	-

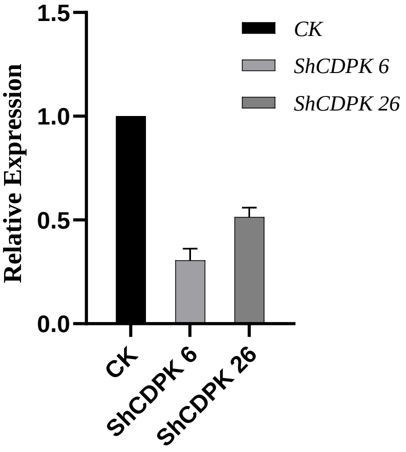

Figure S1. Silence efficiency of ShCDPK6 and ShCDPK26 silenced plants.

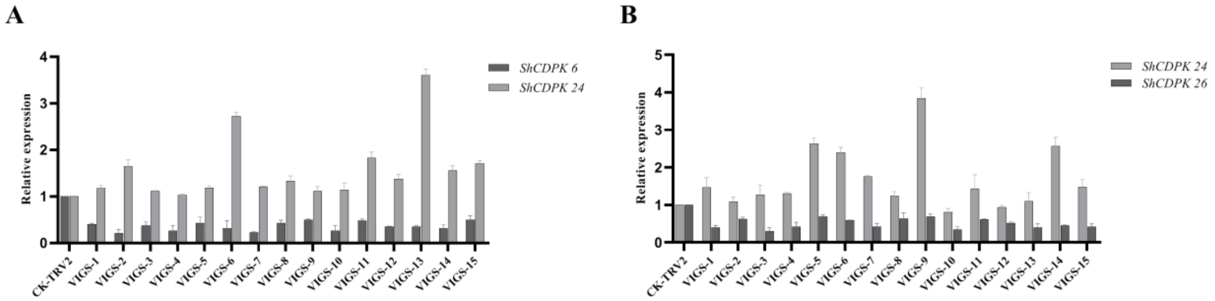

Figure S2. Expression levels of *ShCDPK6* and *ShCDPK26* genes in silenced plants. A: Relative expression of the *ShCDPK6* and *ShCDPK24* genes in *ShCDPK6*-silenced plants; B: Relative expression of the *ShCDPK24* and *ShCDPK26* genes in *ShCDPK26*-silenced plants.

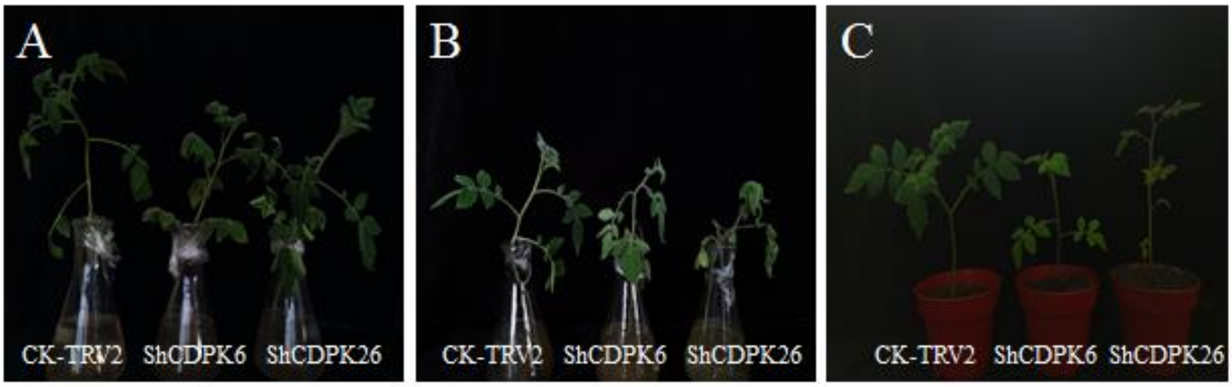

Figure S3. Phenotype of *ShCDPK6* and *ShCDPK26* silenced plants under low temperature(4°C), drought and *Botrytis cinerea* stress. A: Phenotype of *Solanum habrochaites* (CK-TRV2, TRV2-ShCDPK6 and TRV2-ShCDPK26) under low temperature stress; B: Phenotype of *Solanum habrochaites* (CK-TRV2, TRV2-ShCDPK6 and TRV2-ShCDPK26) under drought stress; C: Phenotype of *Solanum habrochaites* (CK-TRV2, TRV2-ShCDPK6 and TRV2-ShCDPK26) under *Botrytis cinerea* stress.
